# Long-term effects of early treatment with SSRIs on cognition and brain development in individuals with 22q11.2 deletion syndrome

**DOI:** 10.1038/s41398-021-01456-x

**Published:** 2021-05-29

**Authors:** Valentina Mancini, Johanna Maeder, Karin Bortolin, Maude Schneider, Marie Schaer, Stephan Eliez

**Affiliations:** 1 Developmental Imaging and Psychopathology Laboratory, Department of Psychiatry, University of Geneva School of Medicine, Geneva, Switzerland; 2 Medical Image Processing Lab, Institute of Bioengineering, EPFL, Lausanne, Switzerland; 3 Clinical Psychology Unit for Developmental and Intellectual Disabilities, Faculty of Psychology and Educational Sciences, University of Geneva, Geneva, Switzerland; 4 Department of Neuroscience, Center for Contextual Psychiatry, Research Group Psychiatry, KU Leuven, Leuven, Belgium; 5 Department of Genetic Medicine and Development, University of Geneva School of Medicine, Geneva, Switzerland

**Keywords:** Schizophrenia, Prognostic markers

## Abstract

Cognitive deficits in individuals at risk of psychosis represent a significant challenge for research, as current strategies for symptomatic treatment are often ineffective. Recent studies showed that atypical cognitive development predicts the occurrence of psychotic symptoms. Additionally, abnormal brain development is known to predate clinical manifestations of psychosis. Therefore, critical developmental stages may be the best period for early interventions expected to prevent cognitive decline and protect brain maturation. However, it is challenging to identify and treat individuals at risk of psychosis in the general population before the onset of the first psychotic symptoms. 22q11.2 deletion syndrome (22q11DS), the neurogenetic disorder with the highest genetic risk for schizophrenia, provides the opportunity to prospectively study the development of subjects at risk for psychosis. In this retrospective cohort study, we aimed to establish if early treatment with SSRIs in children and adolescents with 22q11DS was associated with long-term effects on cognition and brain development. We included 98 participants with a confirmed diagnosis of 22q11DS followed up 2–4 times (age range: 10–32). Thirty subjects without psychiatric disorders never received psychotropic medications, thirty had psychotic symptoms but were not treated with SSRIs, and 38 received SSRIs treatment. An increase in IQ scores characterized the developmental trajectories of participants receiving treatment with SSRIs, even those with psychotic symptoms. The thickness of frontal regions and hippocampal volume were also relatively increased. The magnitude of the outcomes was inversely correlated to the age at the onset of the treatment. We provide preliminary evidence that early long-term treatment with SSRIs may attenuate the cognitive decline associated with psychosis in 22q11DS and developmental brain abnormalities.

## Introduction

Cognitive impairment, including working memory deficits, is increasingly recognized as a core feature of psychosis and plays a crucial role in the overall disability^1,2^. Recent studies have highlighted that cognitive deficits early in development might have far-reaching implications for the emergence of a full-blown psychotic disorder^3,4^. Not only premorbid atypical cognitive development precedes the emergence of the first psychotic symptoms, but it also predicts their later severity^5^. Furthermore, genetic studies demonstrated that part of the total risk variance for schizophrenia is explained by lower IQ and smaller brain volume, suggesting that abnormal brain maturation underlying cognitive decline might represent the earliest expression of the risk for psychosis^3^. Consequently, early interventions expected to prevent cognitive decline are more likely to be effective during postnatal critical developmental stages, as supported by studies in mice^6,7^. However, despite promising translational evidence, to the best of our knowledge, no research in humans has ever considered the impact of early treatment during critical stages of brain development in individuals at risk for psychosis. One of the factors most likely contributing to such lack of studies in humans is the extreme difficulty in identifying individuals at risk for psychosis in the general population before the onset of the first psychotic symptoms, intervene, and follow them up over time.

22q11.2 deletion syndrome (22q11DS) is a neurogenetic disorder characterized by cognitive deficits and high risk for psychosis, with up to 41% of deletion carriers developing a psychotic disorder by adulthood^8^. Studying 22q11DS offers the opportunity to identify critical time windows for intervention and intervene during the premorbid phase of psychosis. The phenomenology of psychotic symptoms and the predictive value of ultra-high risk criteria in 22q11DS are comparable to those of individuals with idiopathic psychosis^9,10^. Moreover, neuroimaging and genetic findings point to a shared neurobiological vulnerability between 22q11DS and idiopathic psychosis^11–13^. Similar to subjects at risk for schizophrenia in the general population, deletion carriers undergo cognitive decline and abnormal brain development before the emergence of psychotic symptoms. A low IQ before the onset of adolescence predates the emergence of psychotic symptoms and cognitive decline—especially in the domain of verbal IQ—is higher in individuals who develop a psychotic disorder^14^. These findings are mirrored by abnormal brain development of cortical and subcortical structures. Deletion carriers with comorbid psychotic symptoms have thinner frontal, temporal and cingulate cortices with altered developmental trajectories of cortical maturation^11,15^. Likewise, many studies indicated that psychosis in 22q11DS is associated with lower volume of frontal and temporal areas^16,17^ and subcortical structures as hippocampus, thalamus, and amygdala^18–20^.

Given these premises, it is evident that individuals with 22q11DS have a remarkably complex neural phenotype, whereby the risk for psychosis is strictly intertwined with cognition and brain maturation. Less clear is how to translate these findings into clinical practice. Currently, there is no gold standard treatment for individuals at risk for psychosis that can ameliorate cognitive abilities and protect neural development^21,22^. Such a treatment would be even more relevant for individuals with 22q11DS, due to the baseline cognitive impairment.

A candidate drug class that may theoretically exert a neuroprotective effect on brain development and attenuate cognitive decline is that of selective serotonin reuptake inhibitor (SSRIs). SSRIs increase serotonin levels by limiting its reuptake at synaptic cleft level with broad effects on action selection, mood, cognition, and learning^23^. The behavioral effects of SSRIs are thought to be mediated by postnatal neurogenesis in the hippocampus and increased brain-derived neurotrophic factor (BDNF) signaling, leading to enhanced neuronal plasticity^24–26^. Chronic administration of SSRIs can reactivate juvenile-like plasticity even after the end of critical developmental periods, suggesting new possibilities for intervention in neurodevelopmental disorders^27–30^.

In human research, growing evidence indicates that treatment with SSRIs can improve cognition and memory performance in psychotic patients, and additionally reduce the burden of negative symptoms in chronic schizophrenia^31–33^. Moreover, successful treatment with SSRIs is accompanied by increased hippocampal volume and frontal and orbitofrontal thickness in patients with major depression^34–36^. Thus, treatment with SSRIs may potentially rescue cognitive decline and abnormal brain development observed in 22q11DS by reducing processes of atrophy and accelerated cortical thinning. Therefore, based on the putative neuroprotective action of SSRIs, the present study aimed to retrospectively investigate the potential effects of long-term SSRIs treatment on cognition and brain development in a longitudinal sample of youths with 22q11DS.

## Materials and methods

### Participants and assessment

At present, the 22q11DS Swiss cohort counts around 200 participants followed-up approximately every 3 years, with a broad age range spanning from 5 to 35 years. All the participants underwent at each visit the administration of neuropsychological testing including the Weschler Adult Intelligence Scale (WAIS-III and WAIS-IV)^37^ or the Weschler Intelligence Scale for Children (WISC-III and WISC-IV)^38^ in order to evaluate general intelligence and reasoning abilities over time and Conner’s Continuous Performance Test (CPT-2 and CPT-3)^39^ to assess attention and impulsivity. Regarding intellectual functioning, over the years to fit the longitudinal design, different versions of the test (version III or IV) were used between participants, but the same version was kept within the participant between visits. To merge different versions of the subtest, we selected measures available in all the batteries, such as Vocabulary, Information, Similarities, Digit Span, Block Design, and Matrix reasoning.

A comprehensive clinical interview using either the Diagnostic Interview for children and adolescents (DICA)^40^ aimed at DSM-IV diagnoses from age 6–18 or the Structured Clinical Interview for DSM-IV axis I Disorders (SCID-II)^41^ and the Structured Interview for Prodromal Syndromes (SIPS)^42^ was performed at each visit by the same psychiatrist (SE). Besides, the Child Behavior Checklist (CBCL)^43^ or the Adult Behavior Checklist (ABCL)^44^ was completed by the parents of the participants. Information regarding the current medications, including the generic name of the drug, the dosage, the onset and the overall length of the treatment were gathered by a trained clinician to have a comprehensive picture of the medication status over time. Additionally, T1-weighted brain scans were acquired at each visit.

### Study design

According to previous studies investigating the effects of SSRIs, we selected three outcome measures: IQ, cortical thickness (CT), and hippocampal volume. We retrospectively examined the medical records of the 22q11DS Swiss cohort and selected individuals with at least one visit before and after the prescription of SSRIs. Details on the types of SSRIs are provided in Table 1.

**Table 1:** Demographic and clinical information of the main groups and subgroups.

	No medication	SSRIs (all subjects)	Psychotic SSRIs	Psychotic No SSRIs
Number of patients	30	38	23	30
Number of visits	91	95	62	78
Number of patients with MRI	27	36	23	26
Number of MRI scans	79	84	60	70
Mean age	19.3 ± 4.7	19.8 ± 5.4	19.4 ± 5.5	19.6 ± 5.9
Sex: *n* females (%)	14 (46.7%)	20 (52.6%)	11 (43.5%)	13 (43.3%)
Mean age at the beginning of SSRIs therapy	N/A	17.9 ± 4.6	17.8 ± 5.4	N/A
Mean duration of SSRIs therapy (years)	N/A	4.2 ± 2.7	4.2 ± 2	N/A
Classes of SSRIs	N/A	*Fluoxetine, n* = *6* *Citalopram, n* = *19* *Escitalopram, n* = *5* *Sertraline, n* = *8*	*Fluoxetine, n* = *4* *Citalopram, n* = *11* *Escitalopram, n* = *1* *Sertraline, n* = *7*	N/A
Mean dosage *(fluoxetine equivalents, mg)*	N/A	26.1 ± 10.6 (10–44)	22.3 ± 8 (10–44)	N/A
Classes of Atypical antipsychotics (AAP)	N/A	*Risperidone, n* = *14* *Aripiprazole, n* = *4* *Amisulpride, n* = *4* *More than 1, n* = *5*	*Risperidone, n* = *14* *Aripiprazole, n* = *4* *Amisulpride, n* = *4* *More than 1, n* = *5*	*Risperidone, n* = *15* *Aripiprazole, n* = *2* *Amisulpride, n* = *5* *More than 1, n* = *3*
Mean dosage *(chlorpromazine equivalents, mg)*	N/A	135.2 ± 80.5 (50-250)	135.2 ± 80.5 (50-250)	141.5 ± 105 (50-250)
*Diagnosis according* *to DSM-IV*	*Major Depression, n* = *0* *Anxiety disorders, n* = *0* *Schizophrenia spectrum disorders, n* = *0*	*Major depression, n* = *27* *Anxiety disorders, n* = *28* *Schizophrenia spectrum disorders, n* = *10*	*Major depression, n* = *18* *Anxiety disorders, n* = *14* *Schizophrenia spectrum disorders, n* = *10*	*Major depression, n* = *0* *Anxiety disorders, n* = *0* *Schizophrenia spectrum disorders, n* = *9*
*Subthreshold psychotic symptoms (SIPS* > *3)*	*n* = *0*	*n* = *13*	*n* = *13*	*n* = *21*

Three age-matched groups of 22q11DS having multiple visits were included:

1. Participants treated with SSRIs (comprising deletion carriers with and without psychotic symptoms);
2. participants without any psychiatric diagnosis nor treatment with psychotropic medications;
3. participants with psychotic symptoms who were never treated with SSRIs.

Although the design of the study is not ideally suited to investigate the effect of a given medication, we have taken several steps to control for confounding variables, comprising the inclusion of subjects with chronic treatment with SSRIs (i.e., length >1.5 years) and the exclusion of individuals with 22q11DS having psychiatric comorbidities other than depression, anxiety and psychotic disorders (such as obsessive-compulsive and autism disorders) in the groups of subjects treated with SSRIs. The presence of any psychiatric condition and any treatment with psychotropic medications was an exclusion criterion for the group without SSRIs treatment. We additionally ensured an age-range spanning from puberty to early adulthood, with no differences across groups. Further details on the inclusion and exclusion criteria are available in the flow chart in the SI.

Written informed consent was obtained from participants and/or their parents if minors. The study was approved by the cantonal ethics committee and conducted according to the Declaration of Helsinki.

### MRI acquisition and analysis

Due to the broad time span of this study, the T1-weighted scans were acquired with two different 3T scanners: a Siemens Trio was used for the first 152 scans and a Siemens Prisma for the remaining 81 scans at the Center for Biomedical Imaging in Geneva. Even though the proportion of scans with each MRI scanner did not differ between the groups tested, the scanner type was entered as covariates in all the analyses in order to avoid confounding factors. The parameters for the acquisition of structural images for the T1-weighted MPRAGE sequence were TR = 2500 ms, TE = 3 ms, flip angle = 8°, acquisition matrix = 256 × 256, field of view = 23.5 cm, voxel size = 0.9 × 0.9 × 1.1 mm and 192 slices.

T1-weighted images underwent fully automated image processing with the software FreeSurfer version 6.0 (https://surfer.nmr.mgh.harvard.edu), comprising skull stripping, intensity normalization, reconstruction of internal and external cortical surfaces and parcellation of subcortical brain regions^45^.

The labeling of hippocampal subfields was obtained by mean of a segmentation technique published with Freesurfer v.6.0^46^, and the quality control of the segmentation was performed as described in detail in our previous study^19^. CT was computed as the shortest distance between the white and the pial cortical surfaces^15,47^. Then, average measures of volume and CT were extracted from 68 regions based on the Desikan parcellation^48^.

### Statistical analyses

#### Mixed effects model analyses

Differences in the developmental trajectories of cognitive and brain measures between the groups were estimated with a mixed model approach (https://github.com/danizoeller/myMixedModelsTrajectories) described in previous studies^19,49^. Briefly, population parameters, such as age and treatment, were modeled as fixed effects and within-subject factors as random effects with the function *nlmefit* in MATLAB R2018a (Mathworks). Developmental trajectories were computed by fitting random slope models to the data, taking into account both within-subject and between-subject effects and the most suitable model order was selected by mean of the Bayesian information criterion. P-values were adjusted for multiple testing with the Benjamini-Hochberg false discovery rate correction (FDR). More details on this statistical approach are available in the SI. Similar to previous studies, we reported FDR corrected p-values and measures of effect size as ß-values for the intercept and the slope in each group^20,50^.

#### Correlation analyses

To test the correlation between the increase of a variable of interest (e.g., IQ or any brain measure) and factors such as the dosage of the drug or the age of the beginning of the therapy we employed the *fitlme* function in MATLAB. The dosage of antipsychotics was calculated using chlorpromazine equivalence for AAP^51^, while employing fluoxetine equivalence for antidepressants^52^. The results were finally covaried for sex, and intracranial volume (ICV) and scan type when indicated and finally adjusted for multiple comparisons.

## Results

### Characteristics of the sample

The overall number of deletion carriers included in the present study was 98 (30 without any medication, 38 treated with SSRIs, of which 23 endorsing psychotic symptoms, and 30 psychotics not treated with SSRIs) with 264 visits and an average of 2.78 visits for each subject (range 2–4). Nineteen deletions carriers received a diagnosis of “schizophrenia and other psychotic disorders” according to DSM-IV, and other 34 subjects presented attenuated psychotic symptoms assessed using the SIPS. Additionally, 28 deletion carriers were diagnosed with major depressive disorders and 23 with anxiety disorders, with 14 subjects having a combination of them. Further details on the demographic and clinical features of the groups are listed in Table 1. Clinical and neuropsychological data were available for all the subjects and all the time-points; however, neuroimaging data, after quality control procedures, were available only for 233 visits (89 subjects).

### Differences in IQ developmental trajectories

Differences in developmental trajectories of cognitive and brain measures were computed across groups and subgroups to explore the potential effect of SSRIs on development.

**Fig. 1:**
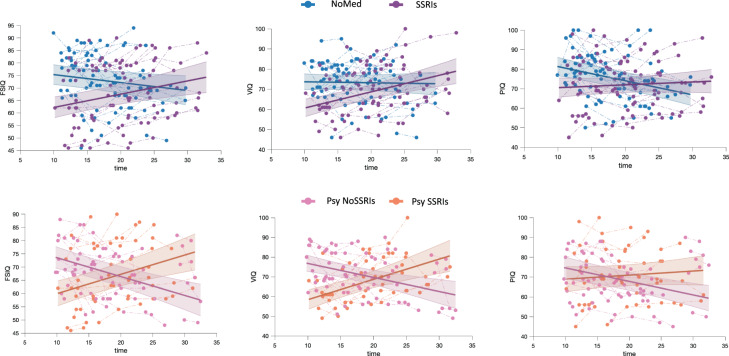
IQ trajectories. Developmental trajectories of full-scale IQ (FSIQ), performance IQ (PIQ) and verbal IQ (VIQ) scores in deletion carriers with and without treatment with SSRIs (upper panel) and deletion carriers endorsing psychotic symptoms with and without treatment with SSRIs (lower panel)).

We first estimated the developmental trajectories of standardized IQ measures comprising Full-Scale IQ (FSIQ), Verbal IQ (VIQ), and Performance IQ (PIQ), obtaining a linear model. Deletion carriers treated with SSRIs exhibited a lower IQ at baseline, but a progressive increase in IQ scores over time with respect to deletion carriers not treated with any medication(FSIQ: 0.53 vs −0.28 points per year; VIQ: 0.15 vs −0.75 points per year; PIQ: 0.89 vs −0.04 points per year; Fig. 1, Table 2).

**Table 2:** Results of the mixed model analyses for IQ, hippocampal volume, cortical volume and CT in deletion carriers without medications compared to deletion carriers treated with SSRIs.

		Group effect				Interaction with age			
	Model order	*p*-value	Log likelihood, df	Intercept NoMed	Intercept SSRIs	*p*-value	Log likelihood, df	Age Slope NoMed	Age Slope SSRIs
*WAIS/WISC*									
FSIQ	Linear	<0.001	26.7, 2	78.5 ± 2.6	56.9 ± 3	<0.001	16.56, 1	−0.32 ± 0.13	0.55 ± 0.15
VIQ	Linear	<0.001	16.18, 2	88.6 ± 3.2	69.1 ± 3.8	<0.001	14.17, 1	−0.75 ± 0.14	0.14 ± 0.16
PIQ	Linear	<0.001	20.17, 2	74.2 ± 2.9	52.6 ± 3.6	<0.001	12.80, 1	−0.05 ± 0.39	0.82 ± 0.17
Vocabulary	Linear	0.013	8.58, 2	7.4 ± 0.9	3.6 ± 1	0.032	4.31, 1	−0.07 ± 0.05	0.09 ± 0.54
*Hippocampal volume*									
Dentate gyrus right	Quadratic	0.028	9.51, 3	312.8 ± 28.4	295.6 ± 25.3	0.046	7.46, 2	−0.28 ± 0.7	−0.01 ± 0.04
CA3 right	Quadratic	0.019	6.89, 3	218.4 ± 27.7	232.3 ± 24.2	0.038	5.36, 2	−0.16 ± 0.9	0.04 ± 0.06
*Cortical thickness*									
Rostral middle frontal left	Linear	0.030	9.8, 2	3.4 ± 0.06	3.2 ± 0.07	0.027	8.30, 1	−0.02 ± 0.003	−0.002 ± 0.003
Rostral middle frontal right	Linear	0.002	12.56, 2	3.3 ± 0.07	2.9 ± 0.08	0.004	9.66, 1	−0.03 ± 0.004	−0.001 ± 0.004
Caudal middle frontal left	Linear	0.030	7.96, 2	3.3 ± 0.06	3.1 ± 0.1	0.020	6.80, 1	−0.03 ± 0.003	−0.001 ± 0.003
Caudal middle frontal right	Linear	0.009	12.12, 2	3.3 ± 0.07	3.0 ± 0.01	<0.001	8.82, 1	−0.03 ± 0.004	−0.001 ± 0.002
Superior frontal left	Linear	0.035	6.66, 2	3.7 ± 0.06	3.5 ± 0.07	0.045	6.39, 1	−0.04 ± 0.003	−0.015 ± 0.003
Superior frontal right	Linear	0.034	6.69, 2	3.6 ± 0.07	3.4 ± 0.07	0.020	7.20, 1	−0.30 ± 0.004	−0.012 ± 0.004
Medial orbitofrontal left	Linear	0.048	4.90, 2	3.5 ± 0.09	3.2 ± 0.09	0.021	4.82, 1	−0.04 ± 0.005	−0.02 ± 0.005
Pars opercularis left	Linear	0.034	11.00, 2	3.4 ± 0.05	3.1 ± 0.06	0.020	9.87, 1	−0.02 ± 0.002	−0.013 ± 0.003
Pars triangularis right	Linear	0.080	7.70, 2	3.4 ± 0.07	3.1 ± 0.08	0.034	7.66, 1	−0.03 ± 0.004	−0.01 ± 0.004
Lateral orbitofrontal right	Linear	0.040	9.78, 2	3.4 ± 0.07	3.2 ± 0.09	0.021	8.91, 1	−0.03 ± 0.004	−0.009 ± 0.001
Superior temporal sulcus left	Linear	0.011	12.98, 2	3.4 ± 0.06	3.1 ± 0.01	0.021	6.81, 1	−0.02 ± 0.003	−0.01 ± 0.003

*P*-values and log-likelihood values with degrees of freedom (df) are provided for the group and the group × age interaction effects. Additionally, β-values for the intercept and the age slope are listed for each group Values for CT are given in mm. All the *p*-values reported are FDR corrected.

Similarly, IQ measures tended to increase over time in deletion carriers with psychotic symptoms treated with SSRIs but not in the group of deletion carriers with psychotic symptoms not treated with SSRIs (FSIQ: 0.73 vs −0.72 points per year; VIQ: 1.03 vs −0.73 points per year; PIQ: 0.23 vs −0.68 points per year; Fig. 1, Table 3).

**Table 3:** Statistically significant results of the mixed model analyses for IQ, hippocampal volume, cortical volume and CT in deletion carriers with psychotic symptoms treated with SSRIs and deletion carriers without psychotic symptoms treated with SSRIs.

		Group effect				Interaction with age			
	Model order	*p*-value	Log likelihood, df	Intercept Psy SSRIs	Intercept Psy NoSSRIs	*p*-value	Log likelihood, df	Age Slope Psy SSRIs	Slope Psy NoSSRIs
*WAIS/WISC*									
FSIQ	Linear	<0.001	34.5, 2	80.6 ± 3	52.6 ± 3.6	<0.001	31.6, 1	0.73 ± 0.17	−0.72 ± 0.15
VIQ	Linear	<0.001	35.9, 2	83.9 ± 3.4	48.1 ± 4.3	<0.001	29, 1	1.03 ± 0.23	−0.73 ± 0.18
PIQ	Linear	0.006	10.27, 2	74.2 ± 2.9	52.6 ± 3.6	<0.001	9.92, 1	0.23 ± 0.2	−0.68 ± 0.17
Hippocampal volume									
CA3 right	Quadratic	0.030	4.67, 3	238.2 ± 24.5	215.3 ± 22.1	0.018	5.86, 2	−0.19 ± 0.7	0.05 ± 0.05
CA4 right	Quadratic	0.049	10.90, 3	252.2 ± 26.3	241.6 ± 21.2	0.027	10.3, 2	−0.18 ± 0.8	0.06 ± 0.16
Whole hippocampus right	Quadratic	0.030	12.2, 3	3057.8 ± 260.3	3009.5 ± 323.3	0.018	12.2, 2	3.32 ± 1.3	−0.86 ± 0.28
Cortical thickness									
Caudal middle frontal right	Linear	0.003	20.1, 2	3.5 ± 0.07	3.0 ± 0.08	<0.001	16.86, 1	−0.01 ± 0.004	−0.04 ± 0.003
Rostral middle frontal right	Linear	0.016	9.42, 2	3.2 ± 0.07	2.8 ± 0.08	0.002	9.02, 1	−0.01 ± 0.003	−0.03 ± 0.003
Pars triangularis right	Linear	0.015	13.49, 2	3.1 ± 0.07	3.3 ± 0.08	0.005	13.52, 1	−0.01 ± 0.005	−0.04 ± 0.004
Precentral gyrus right	Linear	0.016	5.12, 2	2.7 ± 0.08	2.4 ± 0.08	0.003	4.43, 1	−0.003 ± 0.003	−0.01 ± 0.004

*P*-values and log-likelihood values with degrees of freedom (df) are provided for the group and the group × age interaction effects. Additionally, β-values for the intercept and the age slope are listed for each group. Values for CT are given in mm. All the *p*-values reported are FDR corrected.

Among the joint subtests of the WAIS and WISC we found the same pattern of results for the subtests Vocabulary, a measure of lexical knowledge. We additionally tested measures for attention and impulsivity by using CPT (omission errors, commission errors, and hit reaction time), but we did not find any statistically significant difference across groups.

### Differences in developmental trajectories of brain morphometry

**Fig. 2:**
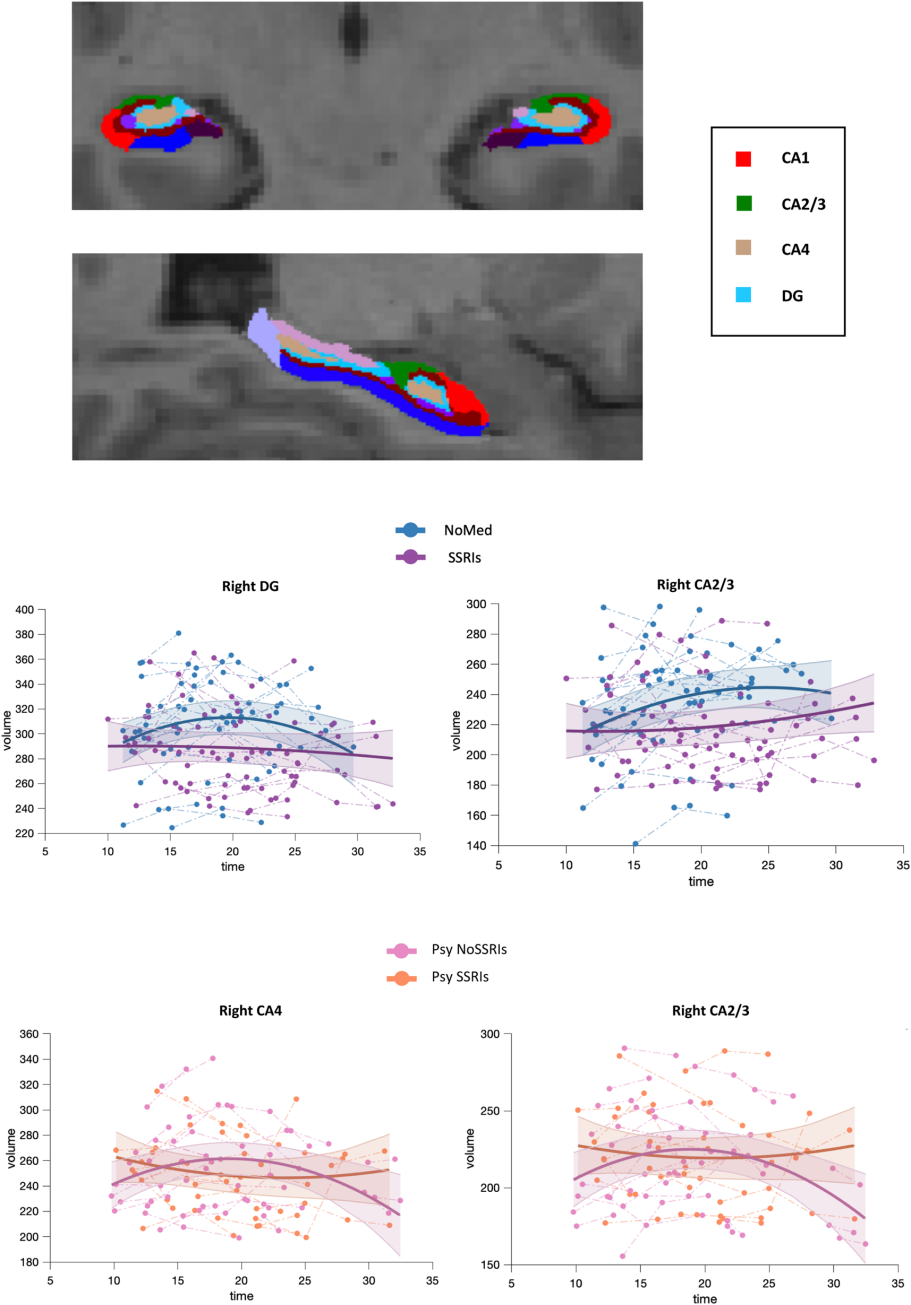
Hippocampal volume trajectories. Developmental trajectories of hippocampal subfields (CA4, CA3, CA4) in deletion carriers with and without treatment with SSRIs (upper panel) and deletion carriers endorsing psychotic symptoms with and without treatment with SSRIs (lower panel)).

We compared the developmental trajectories of the volume of hippocampal subfields across groups and detected a statistically significant difference in right DG and CA3 subfields between deletion carriers treated and not treated with SSRIs (Fig. 2, Table 2) and in right CA3 and CA4 between psychotic deletion carriers treated and not treated with SSRIs (Fig. 2, Table 3). As in prior publications, the trajectories had a second-order model, meaning that the relationship between age and hippocampal volume was quadratic^19,53^.

**Fig. 3:**
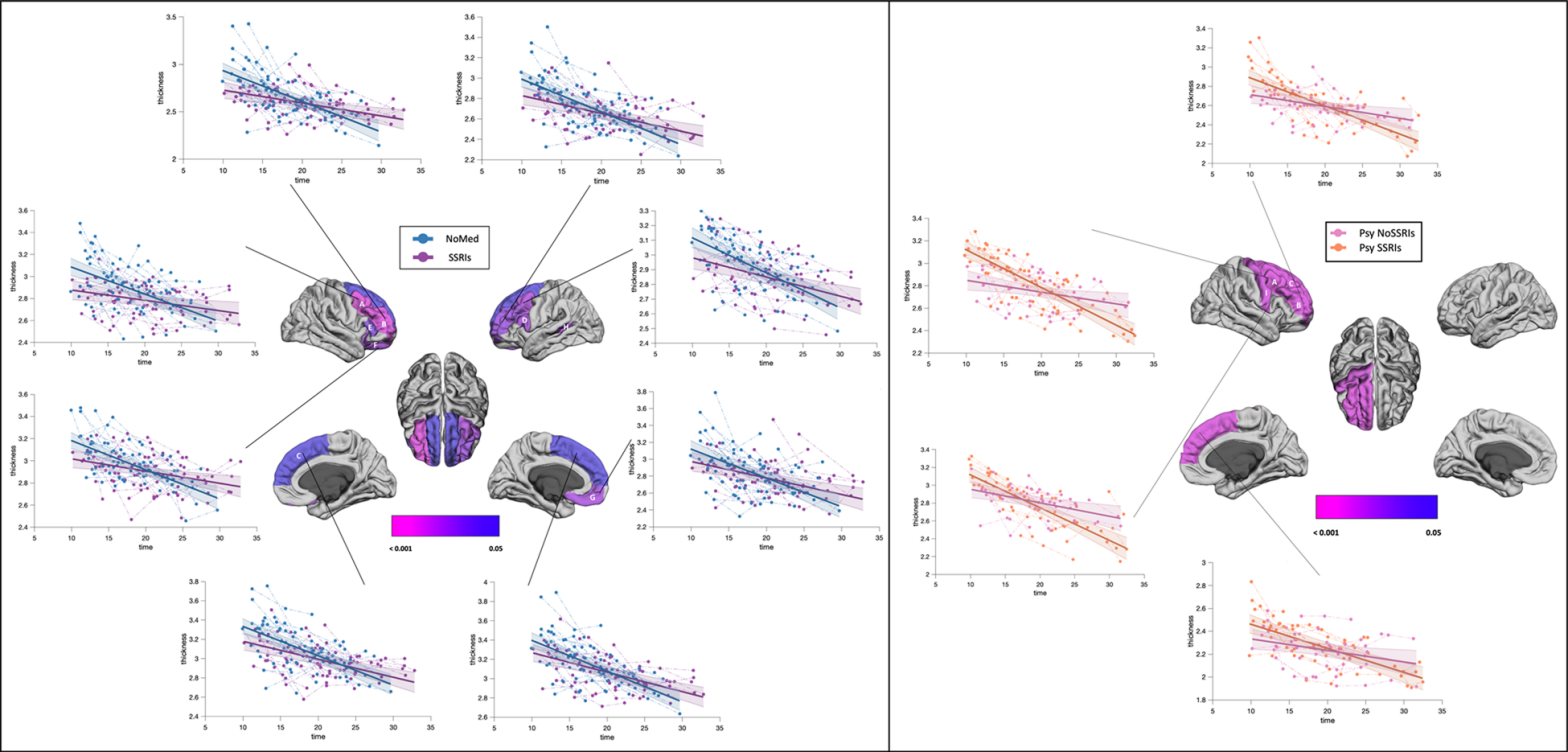
Cortical thickness trajectories. Developmental trajectories of CT of brain regions with divergent maturation in deletion carriers with and without treatment with SSRIs (left panel) and deletion carriers endorsing psychotic symptoms with and without treatment with SSRIs (right panel)). The brain maps are showing regions all the regions with a statistically FDR-corrected significant difference in slope (group × age interaction) between the groups. A = Rostral middle frontal, B = Caudal middle frontal left, C = Superior frontal, D = Pars opercularis, E = Pars triangularis, F = Pars orbitalis, G = lateral orbitofrontal, H = Superior temporal sulcus.

Finally, we tested the divergence of developmental trajectories of cortical thickness (CT), obtaining a linear model. Differences in CT between deletion carriers treated and not treated with SSRIs were found in frontal and temporal regions, whereas CT differences between psychotic deletion carriers treated and not treated with SSRIs were found in frontal regions only (Fig. 3). Overall, volume and CT of individuals not treated with SSRIs tended to decrease over time, in contrast to those treated with SSRIs. Significant FDR corrected *p*-values, log-likelihood ratio for group effect and slope (group × age interaction) and intercept and slope values are listed in Tables 2 and 3. Uncorrected and corrected *p*-values for all the measures tested can be found in the SI (Tables 1, 2).

### Correlations of the outcomes with measures of interest related to the treatment

We further explored the correlation between either the increase in IQ or brain measures and factors potentially contributing to the observed results such as the age of the onset, the dosage, and the duration of the treatment with SSRIs. The increase of each variable was computed as the subtraction between the value at one time-point and the previous one. A negative correlation was found between the age of the onset of SSRIs treatment and VIQ increase (*p*-value = 0.028, *R* = −0.35) and a trend for FSIQ (*p*-value = 0.057, *R* = −0.16). We didn’t find any correlation between the dosage normalized per kg of body weight and duration of the treatment and outcome measures. No statistically significant correlations were found between any of the brain measures and the variables of interest. The only variable correlated to the cognitive outcomes was the age of the beginning of the treatment.

#### Comparison between subgroups treated with SSRIs alone or in combination with atypical antipsychotics

Finally, we explored differences in IQ, CT, and hippocampal volume between deletion carriers treated with SSRIs alone or in combination with atypical antipsychotics (AAP). The results of this exploratory analysis and the relative discussion are available in the SI (Figs. 1–3, Supplementary Table 3, Supplementary Table 4).

## Discussion

To our knowledge, this is the first cohort study exploring the long-term cognitive and neural correlates of early treatment with SSRIs in a population at risk of psychosis. Here we showed that long-term treatment with SSRIs may improve cognitive performances and have a favorable effect on brain development in 22q11.2 deletion carriers.

While all the deletion carriers tend to experience a decrease in IQ from childhood to adulthood, patients with psychotic symptoms are generally characterized by a steeper cognitive decline^14^. Conversely, in our sample, all the patients treated with SSRIs—even those endorsing psychotic symptoms—revealed a relative IQ increase over time. Strikingly, although VIQ has been shown to decrease to a greater extent in psychotic deletion carriers by previous studies^14^, we showed VIQ increase in psychotic deletion carriers treated with SSRIs. When analyzing the WISC/WAIS subscales, we found that these results were mostly driven by subtests related to verbal comprehension and long-term verbal memory.

We additionally investigated the effects of SSRIs treatment on the neural substrates underlying cognition. The SSRIs had a favorable impact on the development of some subfields of the hippocampus, notably DG, CA3, and CA4. Individuals with 22q11DS and comorbid positive psychotic symptoms experience hippocampal atrophy during late adolescence, and DG, CA3, and CA4 are among the most affected subfields^19^. However, our results suggest that treatment with SSRIs may mitigate hippocampal volume loss. Likewise, SSRIs treatment was associated with a potential attenuation of cortical maturation abnormalities related to psychosis. Specifically, our data pointed to a moderate reduction of cortical thinning in frontal areas, especially at the level of the middle frontal cortex.

Overall, these cortical morphometry findings align with previous studies showing the increase of hippocampal volume and thickening of frontal and cingulate regions after SSRIs treatment^34,35^, suggesting that enhanced neurotrophic synthesis may reverse processes of cortical thinning and atrophy even in deletion carriers with psychotic symptoms^54–56^. Therefore, long-term treatment with SSRIs seems to have a selective effect on intellectual functioning and the maturation of a specific network comprising frontal and limbic brain regions. A tentative explanation for our results is that SSRIs might protect brain development from exacerbated volume loss and cortical thinning, possibly ameliorating cognitive functions related to long-term memory.

We tested the potential effects of clinical variables such as the duration, dosage, and age of the onset of the treatment. However, we did not find any significant dose-response relationship. Strikingly, the only factor correlated with the IQ increase was the younger age at the treatment onset. This finding is in line with current neurodevelopmental theories stating that the vulnerability for psychiatric disorders is rooted in the exacerbation of major brain changes normally taking place during adolescence^1,57–60^. Consequently, premorbid interventions during critical developmental periods may be more effective than symptomatic treatment after the emergence of the first clinically relevant symptoms.

Moreover, this interpretation is supported by current research in the field of neuropharmacology. SSRIs have a broad neuroprotective effect, partially depending on the increase of BDNF levels and the enhancement of baseline neurogenesis in the hippocampus, with favorable consequences for neural plasticity^24,61–63^. Although it is well known that the effects of SSRIs in humans are delayed over time, there is new evidence directly linking the activation of the BDNF receptor implied in activity-dependent synaptic plasticity and the maturation of neural circuits to the subsequent clinical effects of antidepressants^64,65^. Additionally, recent studies in mice revealed promising neurodevelopmental outcomes. The administration of SSRIs during adolescence has been shown to improve memory and learning abilities and increase the size of the hippocampus and cortical regions by acting on the number of neurons and dendritic spine density, with long-lasting effects^7^. Therefore, our preliminary results suggest that the neuroprotective effect of SSRIs treatment in individuals at risk for psychosis is potentially greater during adolescence.

### Limitations

Our study comes with several limitations. First, the sample size is not remarkably large; however, considering that 22q11DS is a rare neurogenetic disorder and it is challenging to find longitudinally assessed participants without any medication or selective exposure to a given type of medication, the sample presented in this study is unique. Second, due to the longitudinal nature of our study, the methods employed to assess the population were heterogeneous (i.e., two types of scanner and different versions of tests). Consequently, we added these variables as covariates in all the analyses, and we additionally verified that there were no differences in the rate of each assessment method in each group (Supplementary Table 5).

Third, the design of the study does not allow to control for the confounding factors, as well as other types of studies, such as randomized, double-blind clinical trials. Nonetheless, we have taken several steps to control for confounding factors, as detailed in the SI. Moreover, we analyzed long-term follow-up data, which are extremely rare in medication studies. Future prospective studies are needed to confirm our results.

## Conclusions

In conclusion, we provided preliminary evidence for a neuroprotective effect of SSRIs on cognition and the attenuation of cortical thinning of brain regions implied in higher-order cognitive functions in 22q11DS. As individuals with 22q11DS—especially those endorsing psychotic symptoms—have pervasive cognitive deficits, even moderate reductions of intellectual disability might be relevant. Moreover, despite the high number of comorbidities, antidepressants safety profile in 22q11DS is comparable to that of non-deleted individuals^66^. Therefore, should the results of our study be confirmed in independent cohorts, long-term treatment with low dosages of SSRIs may be indicated, especially in subjects with low IQ.

Finally, our preliminary findings might pave the way for new research lines aimed at exploring the effects of SSRIs in youths at clinical risk for schizophrenia. Early intervention during critical developmental stages may potentially become a promising strategy for individuals with early-life cognitive impairment at risk for psychosis.

## Supplementary information

Supplemental Information
