# Supplementary material for: Long-term effects of early treatment with SSRIs on cognition and brain development in individuals with 22q11.2 deletion syndrome
Source: Transl Psychiatry. 2021 May 29;11:336. doi: 10.1038/s41398-021-01456-x (PMC8164636; doi:10.1038/s41398-021-01456-x)
Supplement: Supplementary file 1 — Supplemental Information [file 41398_2021_1456_MOESM1_ESM.docx]

**Statistical analyses**

*Mixed effects model analyses*

Mixed effects model analyses have been proven to be ideal in modeling multilevel data with a variable time interval and an inconstant age distribution across the visits(1–3).

Population parameters, such as age and treatment, were modeled as fixed effects and within-subject factors as random effects with the function *nlmefit* in MATLAB R2018a (Mathworks). Developmental trajectories were computed by fitting random slope models (constant, linear, quadratic or cubic) to the data, taking into account both within-subject and between-subject effects. The most suitable model order was selected by mean of the Bayesian information criterion, obtaining a full linear model as exemplified by the following equation:

$$Y_{ij}= \beta_{0}+ \beta_{g1}\cdot g_{i}+\beta_{a1}\cdot a_{ij}+ \beta_{ag1}\cdot g_{i}\cdot a_{ij}+ u_{i0}+ u_{i1}\cdot a_{ij}+ \epsilon_{ij}$$

$${Y:variable of interest (e.g. IQ, volume or thickness)}$$

$$i,j:\left[ subjects, scan \right] index$$

$$\beta_{xn}:fixed effects$$

$$g: grouping variable$$

$$a:age$$

$$u:normally distributed random effect$$

$$\epsilon_{i}:normally distributed error term$$

The significance of the between-group differences in the intercept and the slope were evaluated by means of a likelihood ratio test between the full model and any of the following reduced models:

Reduced group effect model

$$Y_{ij}= \beta_{0}+\beta_{a1}\cdot a_{ij}+ u_{i0}+ u_{i1}\cdot a_{ij}+ \epsilon_{ij}$$

Reduced slope model

$$Y_{ij}= \beta_{0}+ \beta_{g1}\cdot g_{i}+\beta_{a1}\cdot a_{ij}+ u_{i0}+ u_{i1}\cdot a_{ij}+\epsilon_{ij}$$

P-values resulting from the likelihood ratio tests refer to the difference in intercept and slope of developmental trajectories of the variables tested between the groups. Finally, p-values were adjusted for multiple testing with the false discovery rate correction (FDR) with the Benjamini-Hochberg method. Confounding factors such as sex, the different version of tests or the type of scanner used to acquire brain images and intracranial brain volume were added to the model as covariates in the respective analyses. Similarly to previous studies, we reported measures of effect size as ß-values for the intercept and the slope in each group (4, 5).

**Sub-analyses on the concomitant treatment with atypical antipsychotics**

The group of patients undergoing chronic SSRIs treatment was divided into 2 subgroups according to the presence (n=18) or absence (n=20) of concomitant therapy with atypical antipsychotics (AAP).

We performed post-hoc exploratory analyses to clarify the contribution of the group treated with SSRIs and AAP by comparing the developmental trajectories in each pair of subgroups (i.e., without medication, with SSRIs only and with SSRIs and AAP treatment).

The increase of the slope of FSIQ, VIQ and PIQ was slightly greater in the group of subjects treated with SSRI and AAP, with respect to both subjects without any medication and treated with SSRIs only (supplementary table 3 and figure 1).

Similar results were found for the volume of some hippocampal subfields such as CA3 and CA4 (supplementary table 3 and figure 2) and for the CT of frontal and temporal regions (supplementary table 4 and figure 3).

**Perspectives on the combination of SSRIs and atypical antipsychotics**

In our cohort, the treatment with SSRIs alone and in association with AAP had a positive effect on the development. However, exploratory analyses revealed that the magnitude of the effects was greater when the two drugs were combined. Although our data do not directly allow us to dissect the molecular mechanism underlying the effects of the combined treatment with SSRIs and AAP, recent studies could help to formulate some hypotheses. For instance, it has been demonstrated that the neurogenic effect of SSRIs is mediated by increased D1 receptors signaling in the DG, and thus can be enhanced by the chronic administration of D1 receptor agonists(6). In support of this notion, long-term D1 receptor agonist treatment improved working memory performance in non-human primates(7). While risperidone does not have a high affinity for D1 receptors, some studies have shown that chronic treatment with risperidone upregulates the expression of D1 receptors only in juvenile rats(8), especially at the level of the hippocampus(9). Previous research in mice has shown that risperidone can rescue a network encompassing ventral hippocampus and prefrontal cortex only during a critical window of time corresponding to late adolescence in humans(10). Similarly, we highlighted that the IQ increase is inversely correlated to the age of treatment’s onset. Therefore, risperidone may act in synergy with SSRIs by increasing the level of D1 receptors in the DG when the treatment starts early in development, potentiating the effect of SSRIs on neurogenesis. On the other hand, SSRIs and AAP may have a complementary rather than synergistic action. This alternative interpretation is supported by previous studies showing that chronic treatment with fluoxetine and clozapine preserves the integrity of GABAergic interneurons in distinct hippocampal subfields(11). Further basic research is needed to test these hypotheses and to explore other potential mechanisms.

**References**

1. Mutlu AK, Schneider M, Debbané M, Badoud D, Eliez S, Schaer M (2013): Sex differences in thickness, and folding developments throughout the cortex. *Neuroimage*. 82: 200–207.

2. Mancini V, Sandini C, Padula MC, Zöller D, Schneider M, Schaer M, Eliez S (2019): Positive psychotic symptoms are associated with divergent developmental trajectories of hippocampal volume during late adolescence in patients with 22q11DS. *Mol Psychiatry*. . doi: 10.1038/s41380-019-0443-z.

3. Dedrick RF, Ferron JM, Hess MR, Hogarty KY, Kromrey JD, Lang TR, *et al.* (2009): Multilevel Modeling: A Review of Methodological Issues and Applications. *Rev Educ Res*. 79: 69–102.

4. Franchini M, Zo D, Ms C, Gentaz E, Glaser B, Wilde HW De, *et al.* (2018): Early Adaptive Functioning Trajectories in Preschoolers With Autism Spectrum Disorders. 1–14.

5. Mancini V, Zöller D, Schneider M, Schaer M, Eliez S (2020): Abnormal development and dysconnectivity of distinct thalamic nuclei in patients with 22q11.2 deletion syndrome experiencing auditory hallucinations. *Biol Psychiatry Cogn Neurosci Neuroimaging*. . doi: 10.1016/j.bpsc.2020.04.015.

6. Shuto T, Kuroiwa M, Sotogaku N, Kawahara Y, Jang J, Yoshinori CS, *et al.* (n.d.): Obligatory roles of dopamine D1 receptors in the dentate gyrus in antidepressant actions of a selective serotonin reuptake inhibitor , fl uoxetine. *Mol Psychiatry*. . doi: 10.1038/s41380-018-0316-x.

7. Castner SA, Williams G V., Goldman-Rakic PS (2000): Reversal of antipsychotic-induced working memory deficits by short-term dopamine D1 receptor stimulation. *Science (80- )*. 287: 2020–2022.

8. Moran-gates T, Grady C, Park YS, Baldessarini RJ, Tarazi FI (2008): Effects of risperidone on dopamine receptor subtypes in developing rat brain. *Eur Neuropsychopharmacol*. 17: 448–455.

9. Santis M De, Lian J, Huang X, Deng C (2016): Early Antipsychotic Treatment in Juvenile Rats Elicits Long-Term Alterations to the Dopamine Neurotransmitter System. *Int J Mol Sci*. 22–27.

10. Mukherjee A, Carvalho F, Eliez S, Caroni P, Mukherjee A, Carvalho F, *et al.* (2019): Long-Lasting Rescue of Network and Cognitive Dysfunction in a Genetic Schizophrenia Model Article Long-Lasting Rescue of Network and Cognitive Dysfunction. *Cell*. 1–16.

11. Filipović D, Stanisavljević A, Jasnić N, Bernardi RE, Inta D, Perić I, Gass P (2018): Chronic Treatment with Fluoxetine or Clozapine of Socially Isolated Rats Prevents Subsector-Specific Reduction of Parvalbumin Immunoreactive Cells in the Hippocampus. *Neuroscience*. 371: 384–394.

22q11DS Swiss Cohort

N = 199

*Inclusion criteria:*

More than one visit

At least 1 visit before 25 years

22q11DS Swiss Longitudinal Cohort

N = 183

**NoMed Group**

N = 30

*Inclusion criteria:*

- Age-matched with SSRIs group
- at least 2 visits

*Exclusion criteria:*

- Any medication to treat psychiatric or neurologic conditions
- Psychiatric diagnosis*

**SSRIs Group**

N = 38

*Inclusion criteria:*

- Chronic therapy with SSRI (>1.5 years)
- at least 1 visit before and after the onset of SSRI therapy

*Exclusion criteria:*

- Medications other than SSRI and AAP

**Psy No SSRIs Group**

N = 30

*Inclusion criteria:*

- Age-matched with SSRIs groups
- at least 2 visits
- psychotic symptoms (SIPS)

*Exclusion criteria:*

- Treatment with SSRIs

SSRIs + AAP

N= 18

*Inclusion criteria:*

therapy with any AAP

SSRIs alone

N= 20

*Exclusion criteria:*

therapy with any AAP

*Including psychotic disorders, depression, anxiety, obsessive-compulsive disorder, attention deficit hyperactivity disorder and autism disorder

**Psy SSRIs Group**

N= 23

*Inclusion criteria:*

Presence of psychotic symptoms irrespectively of therapy with AAP

|  |  | NoMed vs SSRIs | | | | Psy SSRIs vs Psy NoSSRIs | | | |
| --- | --- | --- | --- | --- | --- | --- | --- | --- | --- |
|  | | **Uncorrected** | | **FDR-corrected** | | **Uncorrected** | | **FDR-corrected** | |
|  |  | p-value group | p-value interaction | p-value group | p-value interaction | p-value group | p-value interaction | p-value group | p-value interaction |
| IQ |  |  |  |  |  |  |  |  |  |
| FSIQ |  | < 0.001 | < 0.001 | < 0.001 | **< 0.001** | < 0.001 | < 0.001 | < 0.001 | **< 0.001** |
| VIQ |  | < 0.001 | < 0.001 | < 0.001 | **< 0.001** | < 0.001 | < 0.001 | < 0.001 | **< 0.001** |
| PIQ |  | < 0.001 | < 0.001 | < 0.001 | **< 0.001** | < 0.001 | < 0.001 | < 0.001 | **< 0.001** |
| Hippocampal Volume |  |  |  |  |  |  |  |  |  |
| Tail | left | 0.286 | 0.457 | 0.158 | 0.271 | 0.981 | 0.960 | 0.981 | 0.960 |
| Subiculum | left | 0.800 | 0.800 | 0.637 | 0.784 | 0.108 | 0.049 | 0.145 | 0.065 |
| CA1 | left | 0.398 | 0.530 | 0.786 | 0.786 | 0.322 | 0.194 | 0.354 | 0.239 |
| Molecular layer | left | 0.482 | 0.594 | 0.614 | 0.784 | 0.093 | 0.045 | 0.137 | 0.065 |
| DG | left | 0.362 | 0.526 | 0.724 | 0.786 | 0.074 | 0.031 | 0.132 | 0.063 |
| CA2/3 | left | 0.073 | 0.179 | 0.050 | 0.235 | 0.063 | 0.026 | 0.132 | 0.063 |
| CA4 | left | 0.033 | 0.178 | 0.059 | 0.235 | 0.094 | 0.041 | 0.137 | 0.065 |
| Whole hippocampus | left | 0.660 | 0.704 | 0.754 | 0.786 | 0.037 | 0.014 | 0.132 | 0.057 |
| Tail | right | 0.640 | 0.704 | 0.570 | 0.784 | 0.332 | 0.224 | 0.354 | 0.239 |
| Subiculum | right | 0.098 | 0.196 | 0.116 | 0.244 | 0.041 | 0.019 | 0.132 | 0.062 |
| CA1 | right | 0.113 | 0.200 | 0.122 | 0.244 | 0.074 | 0.031 | 0.132 | 0.063 |
| Molecular layer | right | 0.062 | 0.179 | 0.088 | 0.235 | 0.069 | 0.039 | 0.132 | 0.065 |
| DG | right | 0.003 | **0.028** | 0.007 | **0.046** | 0.218 | 0.211 | 0.268 | 0.239 |
| CA2/3 | right | 0.001 | **0.019** | 0.002 | **0.038** | 0.002 | 0.001 | **0.030** | **0.018** |
| CA4 | right | 0.075 | 0.179 | 0.169 | 0.271 | 0.009 | 0.005 | **0.049** | **0.027** |
| Whole hippocampus | right | 0.078 | 0.179 | 0.073 | 0.235 | 0.004 | 0.002 | **0.030** | **0.018** |

**Table 1.** Results of the mixed model analyses for IQ and hippocampal volume. Uncorrected and FDR-corrected p-values are provided for the group and the group x age interaction effects. (NoMed group= individuals with 22q11DS without medications; SSRIs group= all the individuals with 22q11DS treated with SSRIs; Psy SSRIs group= all the psychotic individuals with 22q11DS treated with SSRIs; Psy NoSSRIs group= the psychotic individuals with 22q11DS not treated with SSRIs).

|  |  | NoMed vs SSRIs | | | | Psy SSRIs vs NoPsy SSRIs | | | |
| --- | --- | --- | --- | --- | --- | --- | --- | --- | --- |
|  | | **Uncorrected** | | **FDR-corrected** | | **Uncorrected** | | **FDR-corrected** | |
|  |  | p-value group | p-value interaction | p-value group | p-value interaction | p-value group | p-value interaction | p-value group | p-value interaction |
| Superior temporal sulcus | right | 0.074 | 0.023 | 0.149 | 0.063 | 0.660 | 0.386 | 0.748 | 0.570 |
| Caudal anterior cingulate | right | 0.031 | 0.031 | 0.091 | 0.072 | 0.709 | N/A | 0.765 | N/A |
| Caudal middle frontal | right | < 0.001 | < 0.001 | **0.009** | **0.001** | < 0.001 | < 0.001 | **0.003** | **0.001** |
| Cuneus | right | 0.402 | 0.199 | 0.543 | 0.315 | 0.201 | 0.082 | 0.325 | 0.281 |
| Entorhinal | right | 0.263 | 0.550 | 0.389 | 0.656 | 0.007 | N/A | 0.043 | N/A |
| Fusiform | right | 0.025 | 0.965 | 0.081 | 0.979 | 0.013 | 0.530 | 0.058 | 0.596 |
| Inferior parietal | right | 0.048 | 0.016 | 0.118 | 0.056 | 0.222 | 0.483 | 0.343 | 0.596 |
| Inferior temporal | right | 0.930 | 0.919 | 0.944 | 0.961 | 0.234 | 0.232 | 0.353 | 0.441 |
| Isthmus cingulate | right | 0.600 | 0.345 | 0.682 | 0.489 | 0.942 | N/A | 0.956 | N/A |
| Lateral occipital | right | 0.327 | 0.223 | 0.463 | 0.345 | 0.056 | 0.242 | 0.152 | 0.441 |
| Lateral orbitofrontal | right | 0.006 | 0.003 | **0.042** | **0.021** | 0.111 | 0.214 | 0.205 | 0.441 |
| Lingual | right | 0.560 | 0.283 | 0.656 | 0.410 | 0.006 | 0.153 | 0.043 | 0.338 |
| Medial orbitofrontal | right | 0.088 | 0.033 | 0.166 | 0.075 | 0.696 | 0.441 | 0.763 | 0.596 |
| Middle temporal | right | 0.112 | 0.127 | 0.195 | 0.227 | 0.058 | 0.611 | 0.152 | 0.653 |
| Parahippocampal | right | 0.663 | 0.688 | 0.704 | 0.793 | 0.033 | N/A | 0.117 | N/A |
| Paracentral | right | 0.167 | 0.060 | 0.264 | 0.124 | 0.256 | 0.115 | 0.370 | 0.325 |
| Pars opercularis | right | 0.024 | 0.009 | 0.081 | 0.048 | 0.462 | 0.275 | 0.561 | 0.473 |
| Pars orbitalis | right | 0.020 | 0.016 | 0.080 | 0.056 | 0.473 | 0.359 | 0.564 | 0.556 |
| Pars triangularis | right | 0.021 | 0.006 | **0.080** | **0.035** | 0.001 | 0.002 | **0.016** | **0.003** |
| Pericalcarine | right | 0.476 | 0.758 | 0.610 | 0.844 | 0.453 | N/A | 0.560 | N/A |
| Postcentral | right | 0.041 | 0.022 | 0.106 | 0.063 | 0.001 | 0.004 | **0.016** | **0.005** |
| Posterior cingulate | right | 0.066 | 0.023 | 0.137 | 0.063 | 0.481 | 0.533 | 0.564 | 0.596 |
| Precentral | right | 0.190 | 0.405 | 0.293 | 0.511 | 0.061 | 0.070 | 0.153 | 0.272 |
| Precuneus | right | 0.032 | 0.022 | 0.091 | 0.063 | 0.023 | 0.149 | 0.096 | 0.338 |
| Rostral anterior cingulate | right | 0.009 | 0.002 | 0.049 | 0.021 | 0.341 | 0.291 | 0.455 | 0.475 |
| Rostral middle frontal | right | < 0.001 | < 0.001 | **0.002** | **0.004** | 0.001 | 0.003 | **0.016** | **0.003** |
| Superior frontal | right | 0.004 | 0.002 | **0.035** | **0.021** | 0.104 | 0.102 | 0.203 | 0.317 |
| Superior parietal | right | 0.028 | 0.026 | 0.085 | 0.064 | 0.067 | 0.538 | 0.158 | 0.596 |
| Superior temporal | right | 0.104 | 0.085 | 0.185 | 0.165 | 0.219 | 0.145 | 0.343 | 0.338 |
| Supramarginal | right | 0.090 | 0.105 | 0.166 | 0.199 | 0.024 | 0.632 | 0.098 | 0.653 |
| Frontal pole | right | 0.407 | 0.185 | 0.543 | 0.306 | 0.854 | N/A | 0.880 | N/A |
| Temporal pole | right | 0.204 | 0.874 | 0.309 | 0.929 | 0.013 | N/A | 0.058 | N/A |
| Transverse temporal | right | 0.059 | 0.157 | 0.134 | 0.266 | 0.684 | 0.524 | 0.762 | 0.596 |
| Insula | right | 0.008 | 0.027 | 0.049 | 0.064 | 0.099 | 0.453 | 0.197 | 0.596 |
| Superior temporal sulcus | left | 0.000 | 0.001 | **0.011** | **0.021** | 0.002 | N/A | 0.022 | N/A |
| Caudal anterior cingulate | left | 0.050 | 0.123 | 0.118 | 0.226 | 0.007 | N/A | 0.043 | N/A |
| Caudal middle frontal | left | 0.002 | 0.001 | **0.030** | **0.020** | 0.990 | 0.887 | 0.990 | 0.887 |
| Cuneus | left | 0.401 | 0.253 | 0.543 | 0.374 | 0.036 | 0.014 | 0.123 | 0.063 |
| Entorhinal | left | 0.050 | 0.666 | 0.118 | 0.781 | 0.002 | N/A | 0.022 | N/A |
| Fusiform | left | 0.121 | 0.707 | 0.205 | 0.802 | 0.004 | N/A | 0.036 | N/A |
| Inferior parietal | left | 0.061 | 0.025 | 0.134 | 0.064 | 0.051 | N/A | 0.145 | N/A |
| Inferior temporal | left | 0.517 | 0.393 | 0.628 | 0.511 | 0.112 | N/A | 0.205 | N/A |
| Isthmus cingulate | left | 0.654 | 0.402 | 0.704 | 0.511 | 0.601 | N/A | 0.692 | N/A |
| Lateral occipital | left | 0.879 | 0.770 | 0.920 | 0.844 | 0.066 | N/A | 0.158 | N/A |
| Lateral orbitofrontal | left | 0.025 | 0.013 | 0.081 | 0.054 | 0.092 | N/A | 0.189 | N/A |
| Lingual | left | 0.553 | 0.375 | 0.656 | 0.511 | 0.033 | N/A | 0.117 | N/A |
| Medial orbitofrontal | left | 0.009 | 0.003 | 0.049 | 0.021 | 0.006 | N/A | 0.043 | N/A |
| Middle temporal | left | 0.006 | 0.007 | 0.044 | 0.045 | 0.045 | N/A | 0.134 | N/A |
| Parahippocampal | left | 0.632 | 0.473 | 0.693 | 0.575 | 0.838 | N/A | 0.876 | N/A |
| Paracentral | left | 0.475 | 0.389 | 0.610 | 0.511 | 0.433 | N/A | 0.554 | N/A |
| Pars opercularis | left | 0.004 | 0.002 | **0.035** | **0.021** | 0.087 | N/A | 0.186 | N/A |
| Pars orbitalis | left | 0.323 | 0.134 | 0.463 | 0.233 | 0.086 | N/A | 0.186 | N/A |
| Pars triangularis | left | 0.040 | 0.014 | 0.106 | 0.055 | 0.045 | 0.009 | 0.134 | 0.045 |
| Pericalcarine | left | 0.913 | 0.999 | 0.941 | 0.999 | 0.039 | N/A | 0.126 | N/A |
| Postcentral | left | 0.157 | 0.063 | 0.254 | 0.126 | 0.088 | N/A | 0.186 | N/A |
| Posterior cingulate | left | 0.484 | 0.230 | 0.610 | 0.348 | 0.256 | N/A | 0.370 | N/A |
| Precentral | left | 0.505 | 0.421 | 0.625 | 0.520 | 0.309 | N/A | 0.438 | N/A |
| Precuneus | left | 0.127 | 0.047 | 0.211 | 0.102 | 0.011 | 0.008 | 0.057 | 0.045 |
| Rostral anterior cingulate | left | 0.962 | 0.801 | 0.962 | 0.865 | 0.342 | N/A | 0.455 | N/A |
| Rostral middle frontal | left | 0.002 | 0.004 | **0.030** | **0.027** | 0.330 | N/A | 0.455 | N/A |
| Superior frontal | left | 0.004 | 0.007 | **0.035** | **0.045** | 0.833 | N/A | 0.876 | N/A |
| Superior parietal | left | 0.018 | 0.010 | 0.080 | 0.048 | 0.163 | N/A | 0.277 | N/A |
| Superior temporal | left | 0.087 | 0.048 | 0.166 | 0.102 | 0.440 | N/A | 0.554 | N/A |
| Supramarginal | left | 0.021 | 0.014 | 0.080 | 0.055 | 0.157 | N/A | 0.274 | N/A |
| Frontal pole | left | 0.613 | 0.947 | 0.683 | 0.976 | 0.374 | N/A | 0.490 | N/A |
| Temporal pole | left | 0.064 | 0.194 | 0.135 | 0.314 | 0.190 | N/A | 0.315 | N/A |
| Transverse temporal | left | 0.601 | 0.378 | 0.682 | 0.511 | 0.144 | N/A | 0.257 | N/A |
| Insula | left | 0.014 | 0.022 | 0.067 | 0.063 | 0.008 | N/A | 0.043 | N/A |

**Table 2.** Results of the mixed model analyses for cortical thickness (CT). Uncorrected and FDR-corrected p-values are provided for the group and the group x age interaction effects. (NoMed group= individuals with 22q11DS without medications; SSRIs group= all the individuals with 22q11DS treated with SSRIs; Psy SSRIs group= all the psychotic individuals with 22q11DS treated with SSRIs; Psy NoSSRIs group= the psychotic individuals with 22q11DS not treated with SSRIs).

|  |  | NoMed vs SSRIs + AAP | | SSRIs only vs SSRIs +AAP | |
| --- | --- | --- | --- | --- | --- |
|  |  | p-value group | p-value interaction | p-value group | p-value interaction |
| IQ |  |  |  |  |  |
| FSIQ |  | **< 0.001** | **0.003** | 0.292 | 0.980 |
| VIQ |  | **< 0.001** | **0.001** | **0.117** | **0.466** |
| PIQ |  | **0.001** | **0.001** | 0.691 | 0.505 |
| Vocabulary |  | **0.040** | **0.030** | **0.158** | **0.330** |
| Information |  | 0.048 | 0.322 | 0.232 | 0.512 |
| Similarities |  | **0.002** | **0.004** | **0.004** | **0.006** |
| Digit Span |  | 0.924 | 0.875 | 0.883 | 0.995 |
| Block design |  | **0.426** | **0.282** | **0.261** | **0.136** |
| Matrix reasoning |  | 0.965 | 0.790 | 0.729 | 0.430 |
| CPT |  |  |  |  |  |
| Omission errors |  | 0778 | 0.637 | 0.809 | 0.563 |
| Comission errors |  | **0.786** | **0.867** | **0.454** | **0.429** |
| Hit reaction time |  | 0.114 | 0.112 | 0.728 | 0.555 |
| Hippocampal Volume |  |  |  |  |  |
| Tail | left | 0.704 | 0.410 | 0.853 | 0.577 |
|  | right | 0.920 | 0.931 | 0.890 | 0.711 |
| Subiculum | left | 0.571 | 0.479 | 0.159 | 0.190 |
|  | right | 0.226 | 0.220 | 0.742 | 0.922 |
| CA1 | left | 0.304 | 0.932 | 0.425 | 0.424 |
|  | right | 0.588 | 0.692 | 0.873 | 0.690 |
| Molecular layer | left | 0.394 | 0.545 | 0.463 | 0.311 |
|  | right | 0.230 | 0.220 | 0.702 | 0.609 |
| DG | left | **0.005** | **0.005** | **0.017** | **0.008** |
|  | right | **0.007** | **0.006** | 0.693 | 0.560 |
| CA2/3 | left | **0.001** | **0.001** | **0.004** | **0.001** |
|  | right | **0.008** | **0.008** | 0.686 | 0.717 |
| CA4 | left | 0.284 | 0.437 | 0.866 | 0.957 |
|  | right | 0.204 | 0.408 | 0.766 | 0.779 |
| Whole hippocampus | left | **0.005** | **0.003** | **0.006** | **0.003** |
|  | right | 0.512 | 0.705 | 0.676 | 0.569 |

Table 3: Results of the mixed model analysis ( p-values for group and group x age interaction) for IQ, CPT, and the volume of hippocampal subfields between subjects without any medication and subjects treated with SSRIs and AAP and between subjects treated only with SSRIs and subjects treated with SSRIs and AAP. (NoMed = No medications; SSRIs only= subjects treated with SSRIs but not AAP; SSRI + AAP = subjects treated with SSRIs and AAP).

|  |  | NoMed vs SSRIs + AAP | | SSRIs only vs SSRIs +AAP | |
| --- | --- | --- | --- | --- | --- |
|  |  | p-value group | p-value interaction | p-value group | p-value interaction |
| Superior temporal sulcus | right | 0.423 | 0.196 | 0.538 | 0.932 |
| Caudal anterior cingulate | right | 0.171 | 0.465 | 0.810 | 0.643 |
| Caudal middle frontal | right | **0.001** | **0.035** | 0.373 | 0.887 |
| Cuneus | right | 0.533 | 0.294 | 0.660 | 0.566 |
| Entorhinal | right | 0.114 | 0.289 | 0.520 | 0.910 |
| Fusiform | right | 0.203 | 0.121 | 0.137 | 0.687 |
| Inferior parietal | right | 0.448 | 0.332 | 0.180 | 0.108 |
| Inferior temporal | right | 0.558 | 0.256 | 0.293 | 0.223 |
| Isthmus cingulate | right | 0.765 | 0.535 | 0.392 | 0.178 |
| Lateral occipital | right | 0.745 | 0.667 | 0.145 | 0.069 |
| Lateral orbitofrontal | right | 0.185 | 0.381 | 0.256 | 0.237 |
| Lingual | right | 0.903 | 0.687 | 0.626 | 0.419 |
| Medial orbitofrontal | right | **0.003** | **0.004** | **0.039** | **0.015** |
| Middle temporal | right | 0.178 | 0.356 | 0.934 | 0.720 |
| Parahippocampal | right | 0.629 | 0.347 | 0.496 | 0.380 |
| Paracentral | right | 0.471 | 0.283 | 0.277 | 0.345 |
| Pars opercularis | right | 0.106 | 0.426 | 0.494 | 0.488 |
| Pars orbitalis | right | 0.772 | 0.760 | 0.582 | 0.333 |
| Pars triangularis | right | 0.422 | 0.208 | 0.352 | 0.266 |
| Pericalcarine | right | 0.941 | 0.541 | 0.102 | 0.061 |
| Postcentral | right | 0.446 | 0.313 | 0.584 | 0.412 |
| Posterior cingulate | right | 0.812 | 0.620 | 0.647 | 0.483 |
| Precentral | right | 0.111 | 0.673 | 0.605 | 0.360 |
| Precuneus | right | 0.511 | 0.758 | 0.443 | 0.328 |
| Rostral anterior cingulate | right | 0.074 | 0.095 | 0.808 | 0.657 |
| Rostral middle frontal | right | **0.003** | **0.002** | 0.417 | 0.259 |
| Superior frontal | right | 0.056 | 0.097 | 0.972 | 0.950 |
| Superior parietal | right | 0.732 | 0.796 | 0.274 | 0.434 |
| Superior temporal | right | 0.583 | 0.866 | 0.650 | 0.463 |
| Supramarginal | right | 0.393 | 0.861 | 0.429 | 0.228 |
| Frontal pole | right | 0.738 | 0.111 | 0.962 | 0.915 |
| Temporal pole | right | **0.029** | 0.156 | 0.317 | 0.268 |
| Transverse temporal | right | 0.166 | 0.704 | 0.519 | 0.259 |
| Insula | right | 0.060 | 0.392 | 0.733 | 0.705 |
| Superior temporal sulcus | left | **0.003** | **0.046** | 0.288 | 0.219 |
| Caudal anterior cingulate | left | 0.125 | **0.042** | 0.258 | 0.234 |
| Caudal middle frontal | left | **0.021** | **0.035** | 0.419 | 0.237 |
| Cuneus | left | 0.964 | 0.787 | 0.622 | 0.551 |
| Entorhinal | left | 0.112 | 0.389 | 0.340 | 0.739 |
| Fusiform | left | 0.119 | 0.412 | 0.741 | 0.627 |
| Inferior parietal | left | 0.708 | 0.439 | 0.317 | 0.481 |
| Inferior temporal | left | 0.273 | **0.004** | 0.691 | 0.567 |
| Isthmus cingulate | left | 0.715 | 0.955 | 0.793 | 0.646 |
| Lateral occipital | left | 0.928 | 0.744 | 0.987 | 0.907 |
| Lateral orbitofrontal | left | 0.301 | 0.337 | 0.720 | 0.646 |
| Lingual | left | 0.802 | 0.667 | 0.651 | 0.788 |
| Medial orbitofrontal | left | **0.049** | 0.212 | 0.134 | 0.827 |
| Middle temporal | left | **0.008** | 0.047 | 0.803 | 0.541 |
| Parahippocampal | left | 0.712 | 0.586 | 0.757 | 0.480 |
| Paracentral | left | 0.859 | 0.624 | 0.324 | 0.311 |
| Pars opercularis | left | 0.147 | 0.116 | 0.786 | 0.884 |
| Pars orbitalis | left | 0.241 | 0.997 | 0.200 | 0.488 |
| Pars triangularis | left | 0.271 | 0.373 | 0.765 | 0.949 |
| Pericalcarine | left | 0.465 | 0.648 | 0.295 | 0.374 |
| Postcentral | left | 0.868 | 0.596 | 0.622 | 0.630 |
| Posterior cingulate | left | 0.898 | 0.912 | 0.906 | 0.663 |
| Precentral | left | 0.504 | 0.548 | 0.466 | 0.220 |
| Precuneus | left | 0.512 | 0.422 | 0.152 | 0.072 |
| Rostral anterior cingulate | left | 0.823 | 0.693 | 0.926 | 0.813 |
| Rostral middle frontal | left | **0.025** | **0.031** | 0.335 | 0.181 |
| Superior frontal | left | 0.205 | 0.200 | 0.961 | 0.945 |
| Superior parietal | left | 0.899 | 0.811 | 0.305 | 0.463 |
| Superior temporal | left | 0.448 | 0.704 | 0.624 | 0.704 |
| Supramarginal | left | 0.259 | 0.218 | 0.999 | 0.977 |
| Frontal pole | left | 0.631 | 0.385 | 0.194 | 0.073 |
| Temporal pole | left | 0.052 | 0.163 | 0.058 | 0.018 |
| Transverse temporal | left | 0.417 | 0.610 | 0.388 | 0.318 |
| Insula | left | 0.075 | 0.548 | 0.639 | 0.680 |

Table 4: Results of the mixed model analysis (p-values for group and group x age interaction) for cortical thickness between subjects without any medication and subjects treated with SSRIs and AAP and between subjects treated only with SSRIs and subjects treated with SSRIs and AAP. (NoMed = No medications; SSRIs only= subjects treated with SSRIs but not AAP; SSRI + AAP = subjects treated with SSRIs and AAP).

|  | NoMed | SSRIs (all subjects) | Psychotic SSRIs | Psychotic No SSRIs | NoMed vs SSRIs | Psy SSRIs vs Psy NoSSRIs |
| --- | --- | --- | --- | --- | --- | --- |
| Number of patients | 30 | 38 | 23 | 30 | N/A | N/A |
| Number of visits | 91 | 95 | 62 | 78 | N/A | N/A |
| Number of WAIS | 59 (64.8%) | 63 (66.3%) | 40 (64.5%) | 45 (57.7%) | 0.832 | 0.412 |
| Number of WISC | 32 (35.2%) | 32 (33.7%) | 22 (33.5%) | 33 (42.3%) |  |  |
| WAIS III | 46 (50.5%) | 47 (49.5%) | 32 (59.3%) | 36 (60.9%) | 0.985 | 0.712 |
| WAIS IV | 13 (14.3%) | 16 (16.8%) | 8 (7.3%) | 9 (4.3%) |  |  |
| WISC III | 24 (26.4%) | 25 (26.3%) | 17 (29.7%) | 22 (26.2%) |  |  |
| WISC IV | 8 (8.8%) | 7 (7.4%) | 5 (3.7%) | 11 (8.6%) |  |  |
| Number of CPT | 72 (79.1%) | 69 (72.6%) | 43 (69.4%) | 48 (61.5%) | 0.301 | 0.335 |
| Number of CPT-3 | 19 (20.9%) | 26 (27.4%) | 19 (30.6%) | 30 (38.5%) |  |  |
| Number of patients with MRI | 27 | 36 | 23 | 26 | N/A | N/A |
| Number of MRI scans | 79 | 84 | 60 | 70 | N/A | N/A |
| Number of MRI scans acquired with 3T Prisma | 54 (68.3%) | 56 (66.7%) | 41 (68.3%) | 42 (60%) | 0.818 | 0.324 |
| Number of MRI scans acquired with 3T Trio | 25 (31.6%) | 28 (33.3%) | 19 (31.7%) | 28 (40%) |  |  |

Table 5: comparison between the different versions of tests and of the different types of MRI scanner used across the 4 groups tested. Chi-square test was employed to assess the differences in percentage for each measure across groups.

**Figure 1:** Developmental trajectories of full-scale IQ (FSIQ), performance IQ (PIQ) and verbal IQ (VIQ) scores in the 3 groups tested (NoMed, SSRIs only and SSRIs + AAP).

**
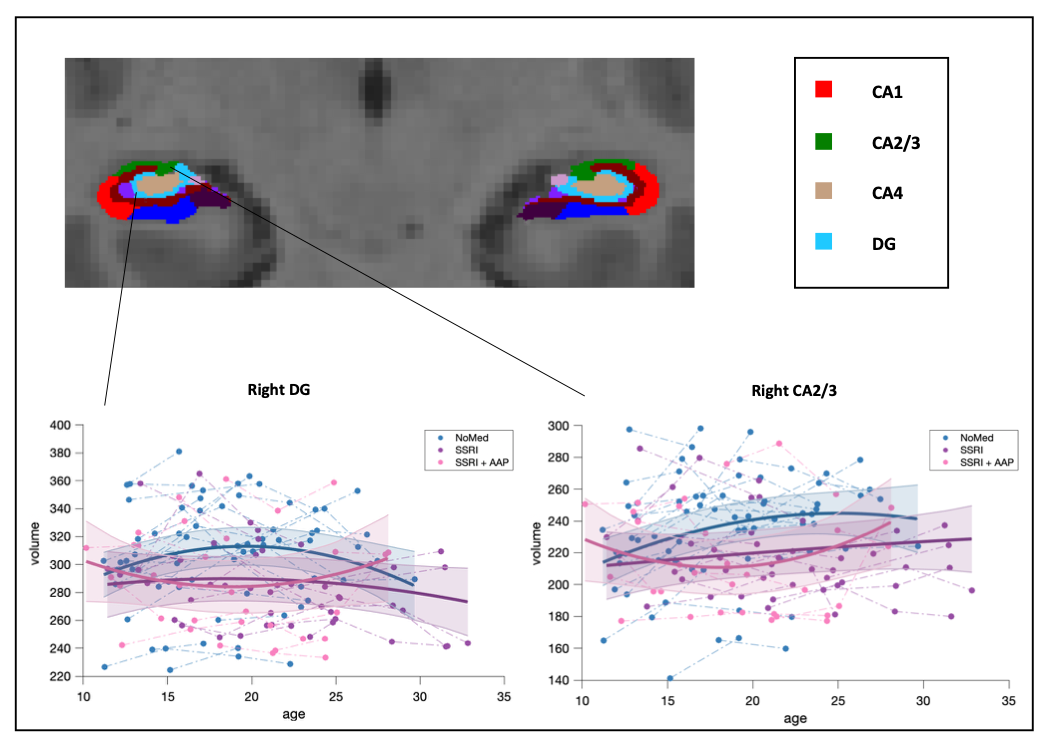
**

**Figure 2:** Developmental trajectories of right dentate gyrus (DG) and CA3 in the 3 groups tested

(NoMed, SSRIs only and SSRIs + AAP).

**Figure 3:** Developmental trajectories of cortical thickness (left panel) and cortical volume (right panel) of brain regions with divergent maturation in the 3 groups tested (NoMed, SSRIs only and SSRIs + AAP).

The brain maps are showing regions with a statistically significant difference in slope (group x age interaction) between the group with and without SSRI medication, to facilitate the comparison with figure 3 in the main text.
